# Risk of cataract and glaucoma among older persons with diabetes in India: a cross-sectional study based on LASI, Wave-1

**DOI:** 10.1038/s41598-023-38229-z

**Published:** 2023-07-24

**Authors:** Junaid Khan, Subhojit Shaw

**Affiliations:** Department of Population and Development, International Institute for Population Sciences, Mumbai, India

**Keywords:** Risk factors, Epidemiology, Diabetes, Neuroendocrine diseases, Eye diseases, Vision disorders

## Abstract

According to the International Diabetes Federation-2019 estimates, India is home to 77 million diabetic individuals which is projected to grow up to 147.2 million by 2045. Diabetes being a progressive health disorder leads to multiple morbidities and complications including eye diseases and visual impairments. As the burden of diabetes mellitus is increasing, eye problems like cataracts and glaucoma are commonly cited problems among the older adults. In this context, this study aims to provide the public health evidences on diabetes associated burden and risk of developing cataracts and glaucoma among older adults aged 60 and above in India. The analytical sample of this cross-sectional study comprised of 31,464 individuals aged 60 and above. Bivariate cross-tabulation and chi-square test were performed to understand the differential in the prevalence of cataracts and glaucoma by diabetes mellitus including the socio-economic and demographic characteristics of the individuals. Binary logistic regression estimation was executed to estimate the adjusted odds ratio for each of the outcome variables within a multivariate framework. The cataract problem affects more than one-fifth of the older people, while glaucoma affects 2% of them. The prevalence of cataract and glaucoma is 29% among diabetic older adults compared to 22% among non-diabetic persons. In terms of gender, the cataract prevalence is comparatively higher among females (25%) than males (21%). It is important to note that while adjusting for socio-economic and demographic characteristics, the likelihood of cataract (AOR 1.495; p-value < 0.01) and glaucoma (AOR 1.554; p-value < 0.01) is significantly higher among older adults with diabetes than among their counterparts. Medical practitioners should conduct prognosis for diabetic eye problems among patients and raise awareness about the potential risks of developing vision loss, such as cataracts and glaucoma, which are more prevalent among individuals with diabetes.

## Introduction

Diabetes mellitus has become one of the major public health issues worldwide due to lifestyle changes, urbanization, limited physical activities, and obesity. The global prevalence of diabetes is projected to rise from 640 million in 2030 to 783 million in 2045^1^. Diabetes is more severe in low and middle-income countries, and it is predicted that by 2025, India will be the world's "diabetes capital," with 69.9 million diabetics^2^. The estimated health expenditure for diabetes ranges from USD 490.1 billion to USD 893.0 billion globally in 2030^3^. However, less than 10% of the global expenditure is spent in low-and middle-income countries and India accounted for less than 1% of the global total expenditure^3^. The impact of diabetes on health ranges from a variety of systemic microvascular complications, such as organ failures, amputations, renal disease, cardiovascular disease, vision loss, and premature death^4^.

Diabetes mellitus and related complications are becoming the leading cause of mortality and morbidity across the world^5^. In particular, diabetes is associated with sensory impairment, including vision and hearing impairment, due to unfavorable glucose-induced inflammation^6,7^. Previous studies indicate that around one-third of individuals with diabetes develop diabetic retinopathy (DR), with approximately one-tenth of those affected experiencing vision-threatening levels of the condition^8^. In India, a study based in 19 cities found that 45 percent of diabetic patients experienced vision loss^9^. A hospital-based study carried out in Maharashtra, India, found that the prevalence of glaucoma among patients is 18.50%^10^. Additionally, diabetes can lead to various ocular complications, including uveitis, diabetic papillopathy, glaucoma, and cataracts^11,12^.

Genetic factors play a significant role in the risk of developing cataracts and glaucoma among older individuals with diabetes^13^. Multiple studies have demonstrated a strong genetic component in the development and progression of these eye conditions^13–15^. Certain genetic variations are associated with an increased susceptibility to cataracts and glaucoma, particularly in individuals with diabetes^13,16^. These genetic factors can influence the structural integrity of the lens and optic nerve, leading to an elevated risk of developing these ocular diseases^15,16^. Understanding the genetic basis of cataracts and glaucoma in diabetic individuals may facilitate early detection, risk assessment, and personalized interventions to mitigate vision loss and improve patient outcomes.

Glaucoma is one of the leading causes of permanent blindness globally^17^. Glaucoma, an optic nerve neurodegenerative disease, results in the death of retinal ganglion cells (RGCs), leading to a loss of vision^4^. Previously, the association between diabetes and glaucoma was debated, but recent research reveals that persons with diabetes are at a higher risk of developing glaucoma^18,19^. Diabetes and hyperglycemia are associated with the glycation of lipids and abnormalities of lipid metabolism, increasing oxidative stress and promoting cellular apoptosis in glaucoma^20^. Similarly, diabetic patients are two times more likely to develop cataracts^21^. The pathogenesis of diabetic cataract has been studied through various pathways, namely increased osmotic stress, oxidative stress or non-enzymatic glycation of lens proteins^9,22^.

The etiology of diabetes-related vision loss problems is linked to hyperglycemia and the duration of diabetes^23^. A retrospective longitudinal study conducted in South Korea revealed that patients with diabetes had a higher likelihood of developing glaucoma compared to non-diabetic individuals, with a hazard ratio of 1.18 among elderly individuals aged 60–79 years^24^. During the year 2020, China and India demonstrated a prevalence of glaucoma at 3.05% and 2.64% respectively within the Asian continent^25^. Both glaucoma and cataracts lead to vision loss without early warning signs or symptoms, and by the time symptoms manifest, the diseases have already progressed significantly. In this regard, a study in India found that just 4.8 percent of patients were aware of glaucoma, and only 3.1 percent had some awareness of the disease^26^. Several studies conducted in hospitals have identified increasing age, high intraocular pressure, hypertension, family history, and diabetes as the primary risk factors closely associated with the development of glaucoma^27–29^. The pathophysiology of cataracts is more pronounced among individuals with diabetes due to the accumulation of deposits in the lenses. A previous study conducted in southern India found a higher prevalence of cataracts in individuals with diabetes (OR 1.55, 95% CI), particularly among women^30^. Non-communicable diseases such as diabetes mellitus, glaucoma, and cataracts have diverse causes including individual's bio-physical, socio-demographic, and behavioral factors. However, prior research conducted in India has been limited in scope. To the best of our knowledge, this study represents the first of its kind to utilize a nationally representative sample survey of adults aged 45 years and above, aiming to explore the association between diabetes and the risk of cataracts and glaucoma among the older population in India.

## Materials and methods

For this study, we used Longitudinal Ageing Study in India (LASI) wave 1 data, which was collected between April 2017 and December 2018 in India as part of the Global Health and Retirement Study (HRS). With the cooperation of the Ministry of Health and Family Welfare, LASI had gathered data on physical and mental health, social security, and family welfare among people aged 45 and higher (MoHFW). The multi-stage stratified probability cluster sampling method was used, in which three-stage and four-stage sampling designs were used for rural and urban areas, respectively. This study used the de-identified data from the LASI, wave 1 survey. The survey received the approval from the Indian Council of Medical Research (ICMR) and the institutional review board held at the International Institute for Population Sciences, Mumbai, India. Informed consent was taken from the participants prior to the survey. The total individual sample size 72,250 respondents. A total of 31,463 (14,930 males and 16,533 females) individuals aged 60 and above constituted the analytical sample of this study. Individuals below the age of 60 were excluded, as the study specifically focused on the population aged 60 and above. The present study intended to examine the diabetes-associated cataract and glaucoma problems among the older population (60+ years) exclusively in India. With the rising prevalence of diabetes and an aging population, understanding these ocular complications can aid in early detection, better management, and improved quality of life, ultimately reducing the burden on healthcare systems.

### Outcome variable

In this study, two major eye or vision problems among older adults were considered. Self-reported questions was asked to the individuals—“Were you diagnosed with an eye or vision problem or condition in one or both eyes?” Those who responded ‘yes’, were further asked “With which problem or condition were you diagnosed?” (a) Presbyopia, (b) Cataract, (c) Glaucoma, (d) Myopia, (e) Hypermetropia, and (f) other. In this study, we selected individuals with cataracts and glaucoma vision problems only.

### Independent (predictor) variable

Self-reported morbidity status was used to estimate the prevalence of diabetes. To determine the state of the disease, respondents were asked to respond to the following question: "Has any health professional ever diagnosed you with diabetes?" The responses were then coded as Yes “1” and No “0” to investigate the association between diabetes as the main predictor of the selected eye problems among older adults in India.

### Control variables

In this study, we adjusted a range of socioeconomic, demographic and lifestyle variables to measure the diabetes associated risk of developing eye problems like cataract and glaucoma among older adults. The control variables were gender (male and female); age (60–69, 70–79 and 80 + years); residence (rural and urban); religion (Hindu, Muslim Christian and others); Indian social class as caste (SC, ST, OBC and none); education (no schooling, completed primary, completed secondary and completed diploma/college); marital status (currently married, widowed, divorced/separated, not married), and monthly per capita consumption expenditure (MPCE) quintile class of the individual (poorest, poorer, middle, richer and richest). 

### Statistical analysis

Univariate statistical analysis was performed to report the descriptive statistics of the study population. To estimate the observed prevalence of cataract and glaucoma by socio-economic and demographic characteristics, the bivariate cross-tabulation analysis was done and the chi-square test was performed to examine the differential across categories of a particular background variable. To estimate the adjusted odds ratio, a binary logistic regression analysis was estimated for each of the outcome variables within a multivariate framework. The standard equation of a logistic model is as follows-

$$log\frac{{p}_{i}}{1-{p}_{i}}=\alpha +\sum_{k=1}^{K}{\beta }_{k}{X}_{ik}$$

Here in the above equation, *pi* denotes the probability that the i-th individual suffers from cataract or glaucoma. STATA version 14 was used to carry out the analysis. The statistical analyses for this study were conducted on a sample that adhered to the defined inclusion and exclusion criteria. The analyses employed a complete case analysis approach, considering the selected variables, and the estimates were adjusted for survey weights.

### Ethics approval and consent to participate

The analysis is based on secondary data available in public domain for research; thus, no approval was required from any institutional review board (IRB). The survey agencies had conducted the field work with prior consent from the respondents.

## Results

### Characteristics of the study population

Table1 shows the descriptive statistics of the study population. A sample of 31,464 older adults aged 60 and above surveyed across India is analysed in this study. About 53% of the total sample is female and 71% of them are from rural areas of India. Eighty-two percent of the respondents belong to the Hindu religion and 19% of them are from the scheduled caste social class. About 57% of them are not educated and only 21% of them completed at least secondary education. Thirty-six percent of the respondents are widowed and 43% of them belong to the lowest two economic quintiles.

**Table 1:** Descriptive statistics of the study variables, India, LASI, 2017–2018.

Variables	Distribution (%)	N
Gender		
Male	47.45	14,930
Female	52.55	16,533
Age		
60–69	58.51	18,409
70–79	30.20	9501
80+	11.29	3552
Residence		
Rural	70.55	22,196
Urban	29.45	9267
Religion		
Hindu	82.22	25,870
Muslim	11.28	3548
Christian	2.86	900
Others	3.64	1144
Caste		
SC	18.91	5948
ST	8.12	2555
OBC	45.23	14,230
None	27.74	8728
Education		
No schooling	56.52	17,782
Completed primary	22.62	7118
Completed secondary	16.80	5285
Completed diploma/college	4.06	1277
Marital status		
Currently married	61.63	19,391
Widowed	36.20	11,388
Divorced/separated	1.00	314
Not married	1.17	369
MPCE quintile		
Poorest	21.70	6829
Poorer	21.71	6831
Middle	20.95	6590
Richer	19.19	6038
Richest	16.45	5175
Diabetics		
No	85.83	26,997
Yes	14.17	4,458
Vision problem with cataract		
No	76.87	24,142
Yes	23.13	7,262
Vision problem with glaucoma		
No	97.56	30,638
Yes	2.44	766
Total		31,464

### Age-sex pattern of cataract and glaucoma among Indian older adults aged 60 and above

**Figure 1:**
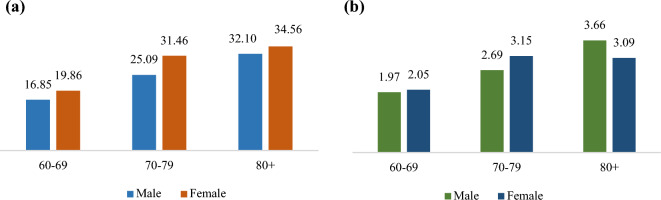
Prevalence of (**a**) cataract and (**b**) glaucoma among older adults by age and sex in India, 2017–18.

The age-sex pattern of cataract and glaucoma is shown in (Fig. 1). From the graph it is observed that the prevalence of cataract shows an increasing pattern with age for both the sexes with females demonstrating higher prevalence of cataract than the males in each age group. For both the sexes, a sharp increase in the prevalence is observed from the 60–69 age group to 70–79 age group. More than one-fourth of the elderly above age 70 in India suffer from the problem of cataract and almost one-third of the female elderly suffer from the problem of cataract. The age-sex pattern of glaucoma prevalence demonstrates an increasing pattern with age among male older adults; whereas, the prevalence shows a drop among females aged 80 years and above.

### Prevalence of cataract among Indian older adults aged 60 and above

More than one-fifth of the older adults suffer from the problem of cataract (Table 2). The male–female pattern shows that the prevalence of cataract is higher among females (24.96%) compared to males (21.10%). The observed prevalence of cataract shows an increasing pattern with age and older adults beyond age 70 carry substantially higher burden of cataract than those in the age group of 60–69. A rural–urban difference is evident and the prevalence of cataract is as high as 29.3% in the urban areas compared to 21% in rural areas (Table 2). Among different religious groups of older persons, Christian older adults show the lowest prevalence (16.63%) of cataract and among different social groups, the older adults from the scheduled tribe class demonstrate the lowest prevalence (13.84%) of cataract. There is no sharp education differential being observed in the prevalence of cataract among Indian older adults. By marital status, individuals who are widowed and those who are divorced or separated demonstrate comparatively higher cataract prevalence of 28% and 30% respectively than others. Economic status does not show any consistent gap in the prevalence of cataract although 22% from the poorest and 23% from the poorer quintile have the problem of cataract. The prevalence of cataract is as high as 29% among those individuals who are diabetic compared to 22% among non-diabetic individuals (Table 2).

**Table 2:** Prevalence of cataract and glaucoma among older adults aged 60 and above by socio-economic and demographic characteristics, India, LASI, 2017–2018.

Predictors	Cataract (%)	X^2^	p-value	Glaucoma (%)	X^2^	p-value
Gender						
Male	21.10	53.1217	0.000	2.38	6.3277	0.012
Female	24.96			2.49		
Age						
60–69	18.44	622.8195	0.000	2.01	32.5038	0.000
70–79	28.35			2.93		
80+	33.42			3.35		
Residence						
Rural	20.54	259.7900	0.000	2.38	0.1333	0.715
Urban	29.31			2.58		
Religion						
Hindu	23.43	308.8575	0.000	2.56	17.3671	0.001
Muslim	23.77			2.12		
Christian	16.63			1.29		
Others	19.47			1.71		
Caste						
SC	22.48	405.0398	0.000	2.64	17.0035	0.001
ST	13.84			2.01		
OBC	23.22			2.16		
None	26.12			2.89		
Education						
No schooling	21.99	44.2999	0.000	2.62	10.3074	0.016
Completed primary	25.56			2.18		
Completed secondary	23.59			2.32		
Completed diploma/college	23.54			1.91		
Marital status						
Currently married	20.36	195.0253	0.000	2.12	16.9551	0.001
Widowed	27.79			3.01		
Divorced/separated	29.90			1.70		
Not married	18.96			2.56		
MPCE quintile						
Poorest	22.20	19.0406	0.001	2.46	17.2548	0.002
Poorer	23.26			2.68		
Middle	22.42			1.79		
Richer	24.24			2.47		
Richest	23.77			2.89		
Diabetics						
No	22.14	215.3452	0.000	2.32	20.3938	0.000
Yes	29.14			3.19		
Total	23.13			2.44		

### Prevalence of glaucoma among Indian older adults aged 60 and above

The prevalence of glaucoma is around 23% among Indian older adults aged 60 and above (Table 2). The male–female pattern of glaucoma prevalence shows no distinct differential. Age wise, individuals in the age group of 70-79 demonstrate 3% prevalence whereas individuals aged 80 and above shows a prevalence of 3.4%. The prevalence of glaucoma in the urban areas is around 2.6% whereas in rural areas it is 2.4%. Older persons from the Hindu religion show a glaucoma prevalence of 2.6% followed by Muslims (a prevalence of 2.1%) and Christians (a prevalence of 1.3%). The caste pattern of prevalence shows that the general category of population carries the highest prevalence (3%) of glaucoma than elderly from other social classes. Education wise, those who are not educated carry the highest prevalence (2.6%) of glaucoma in India (Table 2). While, the marital status pattern of glaucoma prevalence shows that the widowed older adults in India demonstrate the highest burden (3%) of glaucoma prevalence. By economic status, it is observed that the richest class carry the highest prevalence (a prevalence of 2.9%) of glaucoma while the prevalence is observed lowest (a prevalence of 1.8%) among older adults from the middle economic class in India. Certainly, the prevalence of glaucoma is comparatively higher among the diabetic older adults compared to the non-diabetic individuals (Table 2).

### Likelihood of cataract and glaucoma among Indian older adults aged 60 and above

Table 3 shows the estimates of adjusted odds ratios for diabetes and other socio-economic and demographic covariates from the multivariate logistic regression model. It is found that older adults who are diabetic are 1.5 times more likely [AOR 1.495; p-value < 0.01; SE: 0.056] to suffer from cataract problem than those who do not have diabetes when adjusted for other socio-economic and demographic characteristics of the respondents. Female older adults are almost 1.3 times more likely [AOR 1.264; p-value < 0.01; SE: 0.043] to suffer from cataract than males. The estimated odds for different age groups show an increasing pattern and the likelihood is significantly higher in the higher ages. It is estimated that individuals above age 80 are 2.4 [AOR 2.365; p-value < 0.01; SE: 0.108] times more likely to suffer from cataract and individuals in the age group of 70–79 are 1.9 [AOR 1.264; p-value < 0.01; SE: 0.061] times more likely to suffer from eye cataract than those in the age group of 60–69. The religion pattern of estimated odds shows that compared to the Hindu older persons, Christian and others are less likely to suffer from eye cataract. Those who are educated demonstrate higher odds of cataract than those who are not educated.

**Table 3:** Multivariate logistic regression estimation of cataract and glaucoma among older adults aged 60 and above, India, LASI, 2017–18.

	Cataract		Glaucoma	
	Odds ratio	Std. Err	Odds Ratio	Std. Err
Independent (predictor) variable				
Diabetics				
No®				
Yes	1.495***	0.056	1.554***	0.147
Control variables				
Gender				
Male®				
Female	1.264***	0.043	1.082	0.094
Age				
60–69®				
70–79	1.877***	0.061	1.471***	0.121
80+	2.365***	0.108	1.434**	0.171
Residence				
Rural®				
Urban	1.348***	0.043	0.953	0.082
Religion				
Hindu®				
Muslim	1.024	0.046	0.975	0.114
Christian	0.460***	0.032	0.589**	0.103
Others	0.625***	0.046	0.797	0.144
Caste				
SC®				
ST	0.563***	0.035	0.735*	0.110
OBC	0.975	0.042	0.952	0.103
None	0.985	0.045	0.870	0.102
Education				
No schooling®				
Completed primary	1.263***	0.047	0.809*	0.081
Completed secondary	1.264***	0.057	0.881	0.104
Completed diploma/college	1.165*	0.087	0.597*	0.134
Marital status				
Currently married®				
Widowed	1.206***	0.041	1.174	0.102
Divorced/separated	1.143	0.157	1.047	0.380
Not married	0.939	0.116	1.195	0.357
MPCE quintile				
Poorest®				
Poorer	0.967	0.044	1.170	0.138
Middle	0.961	0.044	0.920	0.116
Richer	1.086	0.050	1.317*	0.156
Richest	1.039	0.050	1.466**	0.178

®: reference category; ***p < 0.001, **p < 0.01, *p < 0.05.

**Figure 2:**
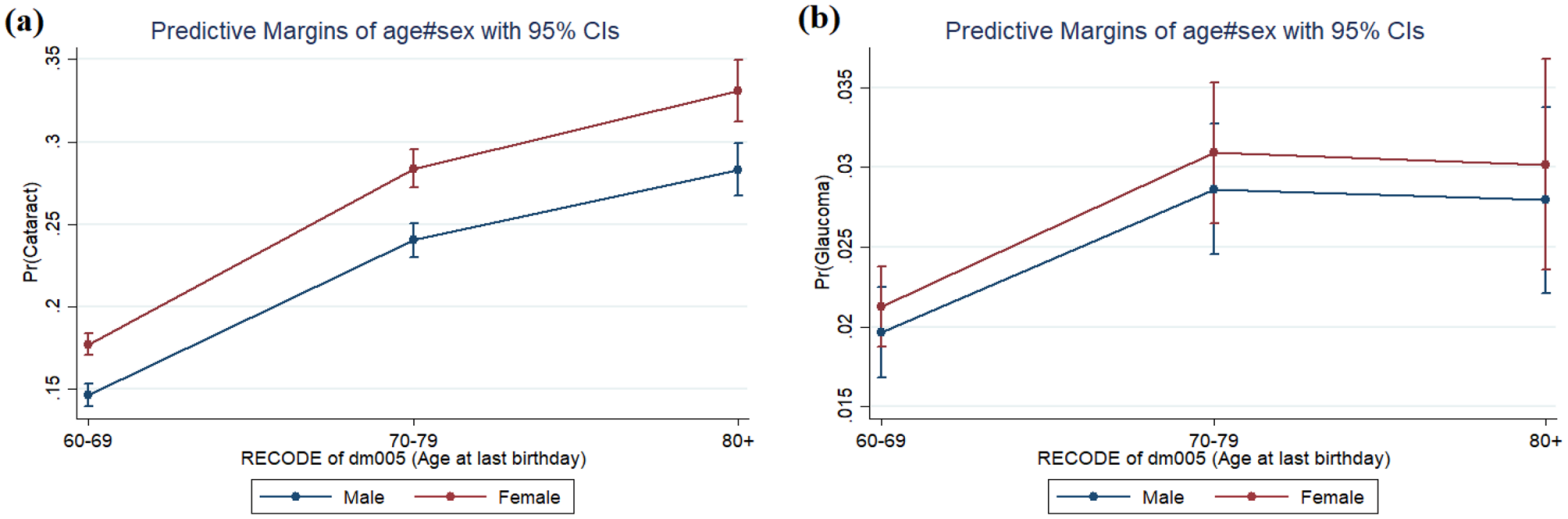
Predicted prevalence of (**a**) cataract and (**b**) glaucoma among older adults with diabetes by age and sex in India, LASI, 2017–2018.

Similar to cataract problem, older adults are also at risk of suffering from glaucoma. The multivariate estimation shows that those who suffer from diabetes are 55% more likely [AOR 1.554; p-value < 0.001; SE: 0.147] to suffer from glaucoma than non-diabetic individuals. Gender does not show any statistically significant association with glaucoma. Age group specific odds hints that persons above age 80 are 1.4 times more likely [AOR 1.434; p-value < 0.001] to experience glaucoma than those in the age group of 60–69. The pattern of adjusted odds ratios and its significance for the rest of the socio-economic and demographic characteristics are shown in Table 3. Adjusted for the control variables, the age-sex classified marginal probabilities are also estimated for both cataract (left panel of the figure) and glaucoma (right panel of the figure) incidence among older adults with diabetes and are shown in (Fig. 2). It is evident that female older adults with diabetes demonstrate a higher risk to suffer from cataract than the male older adults across different age groups.

## Discussion

Diabetes mellitus (DM) as a health condition certainly affects a person’s quality of life^31^. A person who is suffering from DM is vulnerable to different other medical complicacies with a substantial risk to develop different eye problems^32,33^. This study is the first nationally representative, population-based study for India to examine the risk of suffering from cataract and glaucoma among older adults with diabetes. In this study, we investigated the population of older adults aged 60 and above in India to assess the comparative risk of experiencing eye problems like cataract and glaucoma subject to their DM condition. For the generalisability of the findings, we essentially controlled the background characteristics of the study population while estimating the risk of cataract and glaucoma among diabetic older adults compared to non-diabetic. In this context, we compare the prevalence and risk of developing cataract and glaucoma by diabetes status and other socio-economic and demographic characteristics of the study population.

The age-sex pattern of cataract and glaucoma prevalence shows that female older adults demonstrate higher observed prevalence of cataract than males across all age groups and the pattern holds true except in the 80 + age group. The observed prevalence of both the eye problems are higher in the urban areas. A varying pattern of prevalence of cataract and glaucoma is observed across different sub-groups of older adults of different socio-economic and demographic characteristics. For example, the richer MPCE class demonstrates the highest prevalence of cataract whereas the prevalence of glaucoma is observed highest among the richest MPCE class followed by the poorer class. But invariably, the observed prevalence of both the eye problems is comparatively high among the diabetic older adults and diabetes emerged to be a strong predictor of cataract and glaucoma among Indian older adults independent of their socio-economic and demographic characteristics.

In this study, it is found that the burden of cataract and glaucoma is high among females except the prevalence of glaucoma, which is observed higher among 80 + age group of males. A study conducted in Northern India reported equal prevalence of blindness among both the genders^34^. Thus, the risk of blindness persists irrespective of age or sex among diabetic older adults. It has been found that the urban population, people belonging to higher MPCE quintile and upper social class have a higher prevalence of cataract and glaucoma. This result indicates that the population sub-groups with higher burden and risk of developing cataract and glaucoma are the groups identified in this study and are projected to become more prominent in the diabetic capital (India) in the near future. The multivariate estimation confirms that DM is a highly statistically significant predictor of cataract and glaucoma incidence among older adults in India when adjusted for all other socio-economic and demographic characteristics of the individuals.

The prevalence of glaucoma tends to increase with age, and individuals aged over 80 years are at a higher risk^35^. In India, studies have reported varying prevalence rates of glaucoma among the elderly population and the reported prevalence rates depend on factors such as the study population, methodology, and diagnostic criteria used^36–38^. However, it is generally accepted that the prevalence of glaucoma increases with advancing age. As we find in this study, several factors may contribute to the dip in the prevalence of glaucoma burden among individuals aged more than 80 years in India.

Glaucoma is often asymptomatic in the early stages, and older individuals may have a higher likelihood of undiagnosed glaucoma due to limited access to eye care services or lower awareness of the disease^39,40^. As a result, the actual prevalence of glaucoma in this age group may be underestimated. Individuals aged over 80 years may have a higher risk of mortality due to various age-related health conditions^41^. This could lead to a decrease in the number of individuals living with glaucoma in this age group, contributing to the observed dip in prevalence. The dip in prevalence could be influenced by selection bias and the specific study populations included in this study^42,43^. which could result in an underrepresentation of the glaucoma or cataract burden among the oldest age group. Additional factors such as differences in genetic predisposition across different cohorts^44^, socioeconomic status^45^, and access to healthcare services^46^ may also influence the prevalence of glaucoma among individuals aged over 80 years in India. Previous studies show that diabetes is associated with different eye diseases like, retinopathy, cataract and glaucoma primarily; whereby, persons with type-1 diabetes demonstrate higher chances of retinal complications than those with type-2 diabetes^4,32,47–49^. Another study finds that the prevalence of diabetic retinopathy shows a varying prevalence with age and the duration of diabetes mellitus^50^. Different epidemiological studies also report that cataract is a common cause of visual impairment among diabetic patients and diabetic parsons are more likely to develop cataract than non-diabetic persons^51–53^. Also, persons with diabetes are at higher risk of glaucoma^4^. In India, type-2 diabetes associated complications are increasing and diabetic maculopathy, cataract is in subjects with type-2 diabetes burden in the population^54,55^. A study based on India also reports that a prolonged exposure to hyperglycemia is associated with higher risk of cataract among diabetic patients^55^. Increasing age and poor glycemic control are the important risk factors of cataract; whereby, macroalbuminuria and anaemia are the risk factors of cataract among patients with shorter duration of diabetes and among the newly diagnosed diabetic patients^55^. Women older adults demonstrate higher risk of cataract in this study. According to a previous study, difference in the albumin/total protein ratio and serum triglyceride level among women are responsible for higher incidence of cataract among them^56^. Postmenopausal estrogen deficiency among women is also determined to be an important risk factor of cataract among women^57^.

The prevalence of diabetes mellitus and its associated complications are major concerns in diabetes care epidemiology in India^58^. Among various co-morbidities, eye problems are common among diabetic patients^59–61^. Empirical evidences suggest that, Asian Indian people are characterized by high levels of intra-abdominal fat and insulin resistance which prompts them to type-2 diabetes^62^. Although, vision morbidities are unavoidable with progressive age, this study is an exclusive attempt to examine the epidemiological burden of eye problems like cataract and glaucoma among Indian older adults using an exclusive longitudinal ageing survey for India.

Cataract is a common eye condition characterized by clouding of the lens, leading to decreased vision^63^. It is the leading cause of blindness and visual impairment globally, including India^64–66^. While there is no direct evidence linking caste, religion, or marital status to cataract prevalence, certain risk factors can contribute to its development. These risk factors include advancing age, genetics, exposure to ultraviolet radiation, smoking, and certain medical conditions like diabetes^67–69^. Glaucoma is a group of eye diseases characterized by damage to the optic nerve, often associated with increased eye pressure^70,71^. It is also a leading cause of blindness worldwide, including India^72,73^. Similar to cataract, there is limited evidence directly linking caste, religion, or marital status to glaucoma prevalence. However, various risk factors, such as older age, family history, high intraocular pressure, and certain medical conditions like diabetes, can increase the risk of developing glaucoma^70,74–76^.

Diabetes is a chronic metabolic disorder characterized by high blood sugar levels. It can have a significant impact on eye health, potentially leading to various eye conditions, including diabetic retinopathy, cataract, and glaucoma^32,76^. Diabetes affects individuals from diverse backgrounds, including different castes, religions, and marital statuses^77^. However, certain studies have suggested that there might be variations in the prevalence and severity of diabetes across different populations, including differences related to socioeconomic factors and access to healthcare^78^. It's important to note that the impact of caste, religion, and marital status on health conditions can be influenced by a complex interplay of various socioeconomic factors, cultural practices, access to healthcare, and genetic predisposition. Understanding the specific associations between these factors and eye health conditions like cataract and glaucoma would require detailed epidemiological studies that consider a range of variables.

The study highlights that India is home to a significant number of diabetic individuals and the older adults who suffer from diabetes carry a significant risk of developing eye problems like cataract and glaucoma. The study acknowledges that diabetes, a progressive health disorder, can lead to various complications including eye diseases and visual impairments, specifically among older adults aged 60 and above. The study reveals that cataracts affect more than one-fifth of older individuals, while glaucoma affects 2% of them. The study compares the prevalence of cataracts and glaucoma between diabetic and non-diabetic older adults, indicating a higher prevalence among individuals with diabetes. This study notes that the prevalence of cataracts and glaucoma is higher among females compared to males. The estimated adjusted odds ratios indicate a higher likelihood of developing these eye problems compared to their non-diabetic counterparts. The study emphasizes the need for medical practitioners to consider diabetic eye problem prognosis among patients and raise awareness about the potential threats of developing eye problems associated with diabetes. Overall, the study provides public health evidence on the burden and risk of developing cataracts and glaucoma among older adults with diabetes in India, highlighting the importance of addressing these issues to prevent vision loss and improve healthcare outcomes.

The major strength of this study is that it is based upon a large scale nationally representative dataset on older adults in India. The bivariate and multivariate analysis clearly brings out the diabetes associated differential as well as the risk of developing cataract and glaucoma among older adults with diabetes. Additionally, the subgroup specific exploration of the dataset provides the evidence of the public health burden of the eye problems across different population groups in India. Due to cross-sectional nature of the data, we could not provide any argument on the causal association. The dataset does not provide the duration of the diabetes, the individual is actually suffering from, because duration of diabetes is another crucial factor which potentially determines DM associated chronic medical eye complications.

## Conclusion

Complications related to diabetes rank high among the primary causes of blindness in the adult population worldwide. Extensive research has already confirmed the link between diabetes and several eye conditions, such as diabetic retinopathy, cataracts, and glaucoma. The study reveals that older adults in India who have diabetes face an increased risk of developing cataracts and glaucoma compared to the general population. In addition to older adults with diabetes, certain population subgroups in India, including urban older adults and older women who are widowed or divorced/separated, exhibit a higher prevalence of cataract. Given that diabetes is a significant public health concern in India, managing diabetes-related complications, including visual impairments, poses a substantial challenge. In India, diabetes poses a significant public health challenge, particularly concerning the management of comorbidities such as visual impairments. The increased occurrence of cataract and glaucoma in older adults with diabetes, as well as across various population sub-groups, raises concerns regarding the provision of healthcare services. Simultaneously, a significant share of the elderly population resides in rural areas that requires thoughtful intervention for managing glycemic control and addressing the public health burden of associated eye diseases in the rural population. Given the increased risk of cataract and glaucoma associated with diabetes, healthcare professionals should conduct thorough assessments of diabetic eye issues in patients and raise awareness about the potential dangers of developing such problems.

## Data Availability

The datasets used in the study are publicly available and the data request can be put through https://www.iipsindia.ac.in/content/lasi-wave-i.

## Abbreviations

DM: Diabetes mellitus
DR: Diabetic retinopathy
HRS: Global Health and Retirement Study
LASI: Longitudinal Ageing Study in India
MoHFW: Ministry of Health and Family Welfare
MPCE: Monthly per capita consumption expenditure
RGC: Retinal ganglion cells
